# Differential dose–response associations of leisure-time and non-leisure-time physical activity with perceived stress in middle-aged and older adults

**DOI:** 10.1038/s41598-026-52997-4

**Published:** 2026-05-12

**Authors:** Satoshi Seino, Takumi Abe, Yu Nofuji, Tsuneo Konta

**Affiliations:** 1 Institute of Well-Being, Yamagata University, 2-2-2 Iida-Nishi, Yamagata, Yamagata 990-9585 Japan; 2 School of Commerce, Meiji University, 1-9-1 Eifuku, Suginami, Tokyo 168- 8555 Japan; 3 Research Team for Social Participation and Healthy Aging, Tokyo Metropolitan Institute for Geriatrics and Gerontology, 35-2 Sakae, Itabashi, Tokyo 173-0015 Japan; 4 Department of Public Health and Hygiene, Graduate School of Medical Science, Yamagata University, 2-2-2 Iida-Nishi, Yamagata, Yamagata 990- 9585 Japan

**Keywords:** Physical activity, Perceived stress, Domain-specific, Leisure-time, Dose–response, Diseases, Endocrinology, Health care, Risk factors

## Abstract

We examined the dose–response associations of leisure-time and non-leisure-time moderate-to-vigorous physical activity (LT-MVPA and NLT-MVPA, respectively) with high perceived stress among middle-aged and older adults. We cross-sectionally analyzed baseline data from 15,688 adults (6377 men and 9311 women) aged 40–74 years enrolled in the Yamagata Cohort Study. Perceived stress, LT-MVPA, and NLT-MVPA were assessed using questionnaires. Multivariate-adjusted prevalence ratios (PRs) and 95% confidence intervals (CIs) for high perceived stress were estimated using modified Poisson regression. Dose–response relationships were evaluated using restricted cubic splines. In both sexes, LT-MVPA showed an L-shaped association with high perceived stress, with PRs reaching a nadir at approximately 5.0 metabolic equivalent (MET)-hours/day (men: 0.68 [95% CI 0.57–0.82]; women: 0.81 [0.74–0.89]). For NLT-MVPA, men showed a J-shaped association, with the lowest PR at around 15 MET-hours/day (0.78 [0.63–0.96]) and no significant benefit at ≥ 22 MET-hours/day. In contrast, women showed a saturating association, with the highest PR at 20 MET-hours/day (1.27 [1.14–1.42]), remaining elevated at higher levels. LT-MVPA showed a consistent L-shaped association with high perceived stress in both sexes, whereas NLT-MVPA showed sex-specific, non-linear associations. These findings suggest that stress management strategies should account for both MVPA domain and sex.

**Supplementary Information:**

The online version contains supplementary material available at 10.1038/s41598-026-52997-4.

## Introduction

Perceived stress is a psychological and physiological response that individuals commonly experience in everyday life^1^. Its extent is influenced by the frequency and intensity of external stressors and by individuals’ coping abilities^2^. Accumulating evidence indicates that chronic or excessive stress contributes to a wide range of adverse health outcomes, including cardiovascular disease, impaired immune function, and mental health disorders, such as anxiety and depression^3^. In contemporary society, individuals are increasingly exposed to diverse stressors, including occupational demands, strained social relationships, limited supportive networks, financial instability, and rapid technological change^4^. Accordingly, managing stress effectively has become an important component of public health strategies aimed at promoting well-being.

Physical activity (PA), particularly at moderate-to-vigorous intensity, contributes to physical health and mental health, including reduced symptoms of anxiety and depression and enhanced sleep quality^5^. These health benefits were consistently observed for leisure-time PA, including exercise, sports, and recreational activities^6–8^. In contrast, evidence regarding non-leisure-time PA, such as occupational PA, remains inconclusive, with previous studies reporting beneficial^9–11^, adverse^7,12^, mixed^6,13^, or null^8,14–16^ effects on health outcomes. The potentially adverse health effects of PA, particularly in the occupational domain, are referred to as the “PA paradox”^17^. Accordingly, the relationship between moderate-to-vigorous PA (MVPA) and perceived stress may differ by domain: leisure-time MVPA (LT-MVPA) is typically associated with lower perceived stress, while non-leisure-time MVPA (NLT-MVPA) may offer fewer benefits.

However, while extensive evidence supports the physical health benefits of domain-specific MVPA^7,8,12–14^, research on its mental health benefits remains relatively limited^6^, particularly regarding dose–response relationships^18^. For example, a previous meta-analysis using a linear model reported associations between occupational PA and both mental health and mental ill-health^6^. Although this finding highlights the importance of exploring dose–response relationships, to our knowledge, no studies have investigated the dose–response curve shapes of both LT-MVPA and NLT-MVPA in relation to perceived stress. Furthermore, sex differences in the PA paradox have been frequently documented^7,10,12^. Women also report higher levels of perceived stress than that of men^19^, and NLT-MVPA encompasses domains, such as work, transportation, and household tasks—areas where time allocation often differs by sex. Accordingly, sex-stratified analyses are warranted when examining the association between NLT-MVPA and perceived stress.

Thus, we examined the dose–response associations of LT-MVPA and NLT-MVPA with perceived stress among middle-aged and older Japanese adults, focusing on whether the patterns of associations differ by PA domain and sex.

## Methods

### Study participants

This cross-sectional study utilized baseline data collected between 2010 and 2015 from the Yamagata Cohort Study, a community-based prospective cohort study designed to investigate the determinants of health and disease among residents of Yamagata Prefecture, Japan. Details of the study design have been described elsewhere^20,21^. Briefly, adults aged 40–74 years who participated in annual health checkups conducted in seven cities (Yamagata, Kaminoyama, Sakata, Tendo, Higashine, Sagae, and Yonezawa) in Yamagata Prefecture were enrolled. Of the 28,528 individuals who were potentially eligible for the health checkup, 20,969 (8,558 men and 12,411 women) underwent the examination, yielding a participation rate of 73.5%. After excluding 5,281 individuals with missing data on either MVPA or perceived stress, a total of 15,688 participants (6,377 men and 9,311 women) aged 40–74 years were included in the final analysis, yielding a valid participation rate of 55.0%.

### Measurements

#### Perceived stress

Perceived stress was assessed using the question: “How much stress did you experience in the last year?”^19^. Participants responded using one of four options: “strongly felt,” “somewhat felt,” “did not feel much,” or “did not feel at all.” Considering that stress is a common human response^1^, participants who responded “strongly felt” were classified as having high perceived stress, whereas all other responses were categorized as low perceived stress.

#### LT-MVPA and NLT-MVPA

LT-MVPA and NLT-MVPA were assessed using a self-administered PA questionnaire, similar to the one previously used in the baseline survey of the Japan Multi-institutional Collaborative Cohort Study^22,23^.

For LT-MVPA, participants were asked to report the frequency and average duration per session of light (e.g., walking, golf, hiking, recreational gardening, and calisthenics), moderate (e.g., light jogging, swimming, and dancing), and vigorous (e.g., marathon running and competitive sports) activities. The frequency categories (with assigned average times per day) were: almost none (0) 1–3 times/month (0.1), 1–2 times/week (0.2), 3–4 times/week (0.5), and ≥ 5 times/week (0.8). The average duration categories (with assigned hours per activity) were: <30 min (0.3), 30 min to < 1 h (0.8), 1 to < 2 h (1.5), 2 to < 3 h (2.5), 3 to < 4 h (3.5), and ≥ 4 h (4.5). PA intensities were assigned as 3.3, 4.0, and 8.0 metabolic equivalents (METs) for light, moderate, and vigorous activities, respectively^22,23^. The MET-hours/day for each activity category were calculated by multiplying the assigned daily frequency, duration, and intensity. LT-MVPA was estimated by summing the values for light, moderate, and vigorous activities. The LT-MVPA was classified as lowest (0 MET–hours/day), lower-moderate (0.01–1.49 MET-hours/day), upper-moderate (1.50–3.99 MET-hours/day), or highest (≥ 4 MET-hours/day), based on previous studies^22,24^.

For NLT-MVPA, participants were asked to report the average daily time spent in physical hard labor, walking, and standing as part of their usual daily routines, including work, transportation, and household. Time spent on each activity was categorized into one of eight predefined categories (with assigned average hours per day): none (0), < 1 (0.5), 1 to < 3 (2), 3 to < 5 (4), 5 to < 7 (6), 7 to < 9 (8), 9 to < 11 (10), and ≥ 11 (12) h per day. PA intensities were assigned as 4.5, 3.3, and 2.0 METs for physical hard labor, walking, and standing, respectively^23^. NLT-MVPA was estimated by multiplying the daily time spent on physical hard labor and walking by their respective MET values.

Total MVPA (MET-hours/day) was subsequently calculated by summing LT-MVPA and NLT-MVPA. The NLT-MVPA and total MVPA were classified as lowest (0–4.9 MET-hours/day), lower-moderate (5.0–9.9 MET-hours/day), upper-moderate (10.0–19.9 MET-hours/day), or highest (≥ 20.0 MET-hours/day), in compliance with a previous study^23^.

#### Covariates

Based on prior literature regarding PA and mental health^25^, the covariates included age, year of health check-up, area of residence, living situation (living with others or alone), marital status (married, widowed/divorced, or never married), educational attainment (junior high school, high school, junior college/vocational college, or college/graduate school graduation), body mass index (BMI; <18.5, 18.5 − 24.9, or ≥ 25 kg/m^2^), self-rated medical conditions (hypertension, dyslipidemia, heart disease, stroke, diabetes mellitus, and cancer), alcohol drinking and tobacco smoking status (current, never, or former), activities of daily living (ADL) limitation, and sedentary time (< 3, 3 to < 5, 5 to < 7, or ≥ 7 h). BMI was defined as self-rated body weight (kg) divided by self-rated height squared (m^2^). ADL limitation was defined as having difficulty with or being unable to perform any of the following activities: walking approximately 50 m, eating, dressing, bathing, or using the toilet.

#### Statistical analyses

Data were analyzed using Stata version 18.0 (StataCorp, College Station, TX, USA). A two-sided α level of 0.05 was considered statistically significant.

Descriptive statistics were calculated to characterize the study participants. Differences in baseline characteristics between individuals with low and high levels of perceived stress were examined using the unpaired t-test, Mann–Whitney U test, or chi-square test, as appropriate.

For the primary analysis, we used sex-stratified modified Poisson regression models with robust standard errors, treating high perceived stress as the dependent variable and categories of LT-MVPA, NLT-MVPA, or total MVPA as independent variables. In all models, the lowest MVPA category was used as the reference group. Alongside the crude model, two multivariable models were developed. Model 1 was adjusted for age, year of health check-up, area of residence, living situation, marital status, educational attainment, BMI, hypertension, dyslipidemia, heart disease, stroke, diabetes mellitus, cancer, alcohol drinking status, smoking status, and ADL limitation. Model 2 was further adjusted for either NLT-MVPA or LT-MVPA depending on the independent variable, as well as for sedentary time. These models were used to estimate multivariable-adjusted prevalence ratios (PRs) and their corresponding 95% confidence intervals (CIs). In the primary analysis, to reduce potential selection bias, missing values for covariates were treated as a separate category (“missing”) and included in the analysis.

Furthermore, in Model 2, we examined the dose–response relationships between LT-MVPA, NLT-MVPA, or total MVPA and high perceived stress using restricted cubic spline functions. The optimal number of knots—three (10th, 50th, and 90th percentiles), four (5th, 35th, 65th, and 95th percentiles), or five (5th, 27.5th, 50th, 72.5th, and 95th percentiles)—was determined based on the Akaike Information Criterion (AIC), with the model yielding the lowest AIC value being selected^26^. For each spline model, a reference value of 0 MET-hours/day was used.

As a sensitivity analysis, we conducted the same sex-stratified modified Poisson regression models using multiple imputation by chained equations to handle missing covariate data. Twenty imputed datasets were generated, analyzed independently, and subsequently combined for statistical inference using Rubin’s rules^27^. Moreover, because individuals in later life may exhibit lifestyle patterns distinct from those in midlife, we conducted stratified analyses using the same statistical approaches in two age groups: 40–64 years and 65–74 years.

#### Ethics declarations

The study protocol was developed in accordance with the guidelines proposed in the Declaration of Helsinki. Ethical approval for the study was obtained from the Ethics Committee of Yamagata University School of Medicine (approved July 24, 2024; approval number: 2024 − 107). Written informed consent was obtained from all participants prior to enrollment. The study was conducted using an opt-out approach, with information publicly disclosed to allow individuals to decline participation.

## Results

Table 1 presents the characteristics of male participants stratified by perceived stress level. The prevalence of high perceived stress was 12.8%. The medians (interquartile ranges) of the LT-MVPA, NLT-MVPA, and total MVPA were 1.3 (0.3–4.0), 8.9 (3.9–15.6), and 11.0 (4.9–21.6) MET-hours/day, respectively. Compared with individuals with low perceived stress, those with high perceived stress had significantly lower levels of LT-MVPA and total MVPA, were younger, more likely to be never married and current smokers, had longer sedentary time, higher educational attainment, a lower prevalence of hypertension and diabetes mellitus, were less likely to be current drinkers, and had a higher prevalence of ADL limitations.

**Table 1:** Characteristics of male participants, by perceived stress status.

	All		Low stress		High stress		*P*
	(n = 6377)		(n = 5561, 87.2%)		(n = 816, 12.8%)		
LT-MVPA (MET-h/day), median (interquartile range)	1.3	(0.3-4.0)	1.5	(0.3-4.0)	0.8	(0.1–2.5)	< 0.001
NLT-MVPA (MET-h/day), median (interquartile range)	8.9	(3.9–15.6)	8.9	(3.9–15.6)	8.9	(3.9–15.6)	0.363
Total MVPA (MET-h/day), median (interquartile range)	11.0	(4.9–21.6)	11.1	(5.1–21.9)	9.5	(4.4–19.9)	< 0.001
Sedentary time (hours/day), n (%)							< 0.001
< 3	3257	(51.1)	2877	(51.7)	380	(46.6)	
3-4.9	1794	(28.1)	1564	(28.1)	230	(28.2)	
5-6.9	765	(12.0)	660	(11.9)	105	(12.9)	
≥ 7	561	(8.8)	460	(8.3)	101	(12.4)	
Age (years), mean (SD)	64.2	(7.8)	64.8	(7.3)	59.8	(9.5)	< 0.001
40–64, n (%)	2703	(42.4)	2183	(39.3)	520	(63.7)	< 0.001
65–74, n (%)	3674	(57.6)	3378	(60.7)	296	(36.3)	
Year of health check-up, year							0.929
2010, n (%)	1217	(19.1)	1061	(19.1)	156	(19.1)	
2011, n (%)	1352	(21.2)	1189	(21.4)	163	(20.0)	
2012, n (%)	1453	(22.8)	1267	(22.8)	186	(22.8)	
2013, n (%)	869	(13.6)	754	(13.6)	115	(14.1)	
2014, n (%)	594	(9.3)	512	(9.2)	82	(10.0)	
2015, n (%)	892	(14.0)	778	(14.0)	114	(14.0)	
Living alone, n (%)	473	(7.4)	409	(7.4)	64	(7.8)	0.310
Missing	429	(6.7)	384	(6.9)	45	(5.5)	
Marital status, n (%)							< 0.001
Married	5199	(81.5)	4579	(82.3)	620	(76.0)	
Widowed or divorced	406	(6.4)	341	(6.1)	65	(8.0)	
Never married	390	(6.1)	305	(5.5)	85	(10.4)	
Missing	382	(6.0)	336	(6.0)	46	(5.6)	
Educational attainment, n (%)							0.002
Junior high school graduation	1079	(16.9)	976	(17.6)	103	(12.6)	
High school graduation	3458	(54.2)	3012	(54.2)	446	(54.7)	
Junior college/vocational college graduation	617	(9.7)	523	(9.4)	94	(11.5)	
College/graduate school graduation	1024	(16.1)	875	(15.7)	149	(18.3)	
Other/missing	199	(3.1)	175	(3.1)	24	(2.9)	
BMI (kg/m^2^), mean (SD)	23.6	(2.9)	23.6	(2.9)	23.7	(3.1)	0.223
< 18.5, n (%)	223	(3.5)	196	(3.5)	27	(3.3)	
18.5–24.9, n (%)	4303	(67.5)	3765	(67.7)	538	(65.9)	0.467
≥ 25, n (%)	1827	(28.6)	1581	(28.4)	246	(30.1)	
Missing	24	(0.4)	19	(0.3)	5	(0.6)	
Hypertension, n (%)	2376	(37.3)	2112	(38.0)	264	(32.4)	0.008
Missing	163	(2.6)	141	(2.5)	22	(2.7)	
Dyslipidemia, n (%)	849	(13.3)	739	(13.3)	110	(13.5)	0.299
Missing	32	(0.5)	25	(0.4)	7	(0.9)	
Heart disease, n (%)	369	(5.8)	324	(5.8)	45	(5.5)	0.786
Missing	17	(0.3)	14	(0.3)	3	(0.4)	
Stroke, n (%)	245	(3.8)	220	(4.0)	25	(3.1)	0.458
Missing	9	(0.1)	8	(0.1)	1	(0.1)	
Diabetes mellitus, n (%)	721	(11.3)	651	(11.7)	70	(8.6)	0.014
Missing	82	(1.3)	75	(1.3)	7	(0.9)	
Cancer, n (%)	375	(5.9)	327	(5.9)	48	(5.9)	0.889
Missing	39	(0.6)	33	(0.6)	6	(0.7)	
Alcohol drinking status (current), n (%)	5079	(79.6)	4444	(79.9)	635	(77.8)	0.044
Missing	19	(0.3)	19	(0.3)	0	(0)	
Smoking status (current), n (%)	1455	(22.8)	1225	(22.0)	230	(28.2)	0.001
Missing	71	(1.1)	64	(1.2)	7	(0.9)	
ADL limitation, n (%)	193	(3.0)	155	(2.8)	38	(4.7)	0.012
Missing	207	(3.2)	178	(3.2)	29	(3.6)	

ADL, activities of daily living; BMI, body mass index; LT, leisure-time; METs, metabolic equivalents; MVPA, moderate-to-vigorous physical activity; NLT, non-leisure-time; SD, standard deviation.

The characteristics of female participants stratified by perceived stress level are shown in Table 2. The prevalence of high perceived stress was 28.6%. The medians (interquartile ranges) of the LT-MVPA, NLT-MVPA, and total MVPA were 1.0 (0.2–2.6), 8.9 (3.9–15.6), and 9.7 (4.4–18.2) MET-hours/day, respectively. Compared with individuals with low perceived stress, those with high perceived stress had significantly lower levels of LT-MVPA, were younger, more likely to be living alone, current drinkers, current smokers, had higher educational attainment, lower BMI, and a lower prevalence of hypertension.

**Table 2:** Characteristics of female participants, by perceived stress status.

	All		Low stress		High stress		*P*
	(n = 9311)		(n = 6652, 71.4%)		(n = 2659, 28.6%)		
LT-MVPA (MET-h/day), median (interquartile range)	1.0	(0.2–2.6)	1.1	(0.3–2.9)	0.8	(0.1–2.3)	< 0.001
NLT-MVPA (MET-h/day), median (interquartile range)	8.9	(3.9–15.6)	8.9	(3.9–15.6)	8.9	(3.9–15.6)	0.282
Total MVPA (MET-h/day), median (interquartile range)	9.7	(4.4–18.2)	9.7	(4.5–18.4)	9.5	(4.4–18.1)	0.615
Sedentary time (hours/day), n (%)							0.616
< 3	3945	(42.4)	2847	(42.8)	1098	(41.3)	
3-4.9	3103	(33.3)	2203	(33.1)	900	(33.8)	
5-6.9	1411	(15.2)	998	(15.0)	413	(15.5)	
≥ 7	852	(9.2)	604	(9.1)	248	(9.3)	
Age (years), mean (SD)	61.3	(8.5)	62.3	(8.2)	58.6	(8.8)	< 0.001
40–64, n (%)	5564	(59.8)	3627	(54.5)	1937	(72.8)	< 0.001
65–74, n (%)	3747	(40.2)	3025	(45.5)	722	(27.2)	
Year of health check-up, year							0.653
2010, n (%)	1893	(20.3)	1343	(20.2)	550	(20.7)	
2011, n (%)	2020	(21.7)	1437	(21.6)	583	(21.9)	
2012, n (%)	2269	(24.4)	1606	(24.1)	663	(24.9)	
2013, n (%)	1238	(13.3)	884	(13.3)	354	(13.3)	
2014, n (%)	841	(9.0)	614	(9.2)	227	(8.5)	
2015, n (%)	1050	(11.3)	768	(11.5)	282	(10.6)	
Living alone, n (%)	747	(8.0)	572	(8.6)	175	(6.6)	0.001
Missing	540	(5.8)	401	(6.0)	139	(5.2)	
Marital status, n (%)							0.175
Married	7388	(79.3)	5270	(79.2)	2118	(79.7)	
Widowed or divorced	1262	(13.6)	917	(13.8)	345	(13.0)	
Never married	218	(2.3)	143	(2.1)	75	(2.8)	
Missing	443	(4.8)	322	(4.8)	121	(4.6)	
Educational attainment, n (%)							< 0.001
Junior high school graduation	1075	(11.5)	865	(13.0)	210	(7.9)	
High school graduation	5035	(54.1)	3590	(54.0)	1445	(54.3)	
Junior college/vocational college graduation	2494	(26.8)	1698	(25.5)	796	(29.9)	
College/graduate school graduation	511	(5.5)	354	(5.3)	157	(5.9)	
Other/missing	196	(2.1)	145	(2.2)	51	(1.9)	
BMI (kg/m^2^), mean (SD)	22.5	(3.3)	22.6	(3.3)	22.3	(3.4)	< 0.001
< 18.5, n (%)	788	(8.5)	509	(7.7)	279	(10.5)	
18.5–24.9, n (%)	6594	(70.8)	4730	(71.1)	1864	(70.1)	< 0.001
≥ 25, n (%)	1875	(20.1)	1372	(20.6)	503	(18.9)	
Missing	54	(0.6)	41	(0.6)	13	(0.5)	
Hypertension, n (%)	2424	(26.0)	1791	(26.9)	633	(23.8)	0.004
Missing	182	(2.0)	136	(2.0)	46	(1.7)	
Dyslipidemia, n (%)	1602	(17.2)	1148	(17.3)	454	(17.1)	0.815
Missing	46	(0.5)	31	(0.5)	15	(0.6)	
Heart disease, n (%)	220	(2.4)	155	(2.3)	65	(2.4)	0.902
Missing	16	(0.2)	12	(0.2)	4	(0.2)	
Stroke, n (%)	143	(1.5)	95	(1.4)	48	(1.8)	0.407
Missing	10	(0.1)	7	(0.1)	3	(0.1)	
Diabetes mellitus, n (%)	417	(4.5)	301	(4.5)	116	(4.4)	0.110
Missing	82	(0.9)	67	(1.0)	15	(0.6)	
Cancer, n (%)	538	(5.8)	383	(5.8)	155	(5.8)	0.937
Missing	59	(0.6)	41	(0.6)	18	(0.7)	
Alcohol drinking status (current), n (%)	3535	(38.0)	2448	(36.8)	1087	(40.9)	< 0.001
Missing	195	(2.1)	152	(2.3)	43	(1.6)	
Smoking status (current), n (%)	459	(4.9)	288	(4.3)	171	(6.4)	< 0.001
Missing	454	(4.9)	355	(5.3)	99	(3.7)	
ADL limitation, n (%)	374	(4.0)	269	(4.0)	105	(3.9)	0.717
Missing	254	(2.7)	187	(2.8)	67	(2.5)	

ADL, activities of daily living; BMI, body mass index; LT, leisure-time; METs, metabolic equivalents; MVPA, moderate-to-vigorous physical activity; NLT, non-leisure-time; SD, standard deviation.

The multivariate-adjusted PRs and 95% CIs of LT-MVPA, NLT-MVPA, and total MVPA for high perceived stress in men are presented in Table 3. Compared with the lowest LT-MVPA group, both the upper-moderate (PR, 0.71; 95% CI, 0.58–0.87) and highest (PR, 0.68; 95% CI, 0.55–0.84) LT-MVPA groups exhibited significantly lower PRs for high perceived stress, even in the Model 2 (*P* for trend = 0.013). For NLT-MVPA and total MVPA, only the upper-moderate group exhibited significantly lower PRs than the lowest group (NLT-MVPA: PR, 0.82; 95% CI, 0.68–0.99; total MVPA: PR, 0.81; 95% CI, 0.68–0.97).

**Table 3:** PRs (95% CIs) of LT-MVPA, NLT-MVPA, and total MVPA for high perceived stress in men.

Variables	Number of cases per participants	Prevalence (%)	Crude				Model 1				Model 2		
			PR	(95% CI)	*P*		PR	(95% CI)	*P*		PR	(95% CI)	*P*
LT-MVPA categories (MET-hours/day)													
Lowest (0)	198/1022	19.4	1.00	(Ref.)			1.00	(Ref.)			1.00	(Ref.)	
Lower-moderate (0.01–1.49)	329/2310	14.2	0.74	(0.63–0.86)	< 0.001		0.87	(0.74–1.02)	0.080		0.87	(0.74–1.02)	0.086
Upper-moderate (1.50–3.99)	159/1578	10.1	0.52	(0.43–0.63)	< 0.001		0.71	(0.58–0.87)	0.001		0.71	(0.58–0.87)	0.001
Highest (≥ 4.0)	130/1467	8.9	0.46	(0.37–0.56)	< 0.001		0.68	(0.55–0.84)	< 0.001		0.68	(0.55–0.84)	< 0.001
	816/6377	12.8	Trend		< 0.001		Trend		0.018		Trend		0.013
NLT-MVPA categories (MET-hours/day)													
Lowest (0-4.9)	368/2534	14.5	1.00	(Ref.)			1.00	(Ref.)			1.00	(Ref.)	
Lower-moderate (5.0-9.9)	132/1200	11.0	0.76	(0.63–0.91)	0.004		0.86	(0.71–1.03)	0.097		0.89	(0.74–1.07)	0.219
Upper-moderate (10.0-19.9)	140/1311	10.7	0.74	(0.61–0.88)	0.001		0.77	(0.64–0.93)	0.005		0.82	(0.68–0.99)	0.040
Highest (≥ 20.0)	176/1332	13.2	0.91	(0.77–1.08)	0.267		0.95	(0.81–1.13)	0.575		1.04	(0.87–1.23)	0.667
	816/6377	12.8	Trend		0.836		Trend		0.634		Trend		0.149
Total MVPA categories (MET-hours/day)													
Lowest (0-4.9)	251/1605	15.6	1.00	(Ref.)			1.00	(Ref.)			1.00	(Ref.)	
Lower-moderate (5.0-9.9)	173/1267	13.7	0.87	(0.73–1.04)	0.138		1.01	(0.85–1.20)	0.914		1.01	(0.85–1.21)	0.872
Upper-moderate (10.0-19.9)	191/1801	10.6	0.68	(0.57–0.81)	< 0.001		0.79	(0.66–0.95)	0.010		0.81	(0.68–0.97)	0.019
Highest (≥ 20.0)	201/1704	11.8	0.75	(0.63–0.90)	0.001		0.87	(0.73–1.03)	0.110		0.91	(0.76–1.08)	0.280
	816/6377	12.8	Trend		0.228		Trend		0.757		Trend		0.824

Model 1: Adjusted for age, year of health check-up, area of residence, living situation, marital status, educational attainment, body mass index, hypertension, dyslipidemia, heart disease, stroke, diabetes mellitus, cancer, alcohol drinking status, smoking status, and activities of daily living limitation.

Model 2: Adjusted for variables in Model 1 plus sedentary time. For LT-MVPA, further adjusted for NLT-MVPA. For NLT-MVPA, further adjusted for LT-MVPA.

CI, confidence interval; LT, leisure-time; METs, metabolic equivalents; MVPA, moderate-to-vigorous physical activity; NLT, non-leisure-time; PR, prevalence ratio.

The results of the sensitivity analysis using multiple imputation for missing covariate data were consistent with those of the primary analysis (Supplementary Table 1). Stratified analyses by age group showed similar results to those of the primary analysis among participants aged 40–64 years (Supplementary Table 2), whereas no significant associations were observed between NLT-MVPA or total MVPA and high perceived stress in the 65–74-year age group (Supplementary Table 3).

Table 4 presents the multivariate-adjusted PRs and 95% CIs of LT-MVPA, NLT-MVPA, and total MVPA for high perceived stress in women. Compared with the lowest LT-MVPA group, the lower-moderate (PR, 0.88; 95% CI, 0.81–0.95), upper-moderate (PR, 0.80; 95% CI, 0.72–0.88), and highest (PR, 0.78; 95% CI, 0.69–0.87) LT-MVPA groups exhibited significantly lower PRs for high perceived stress, even in the Model 2 (*P* for trend = 0.001). Conversely, compared with the lowest NLT-MVPA group, the upper-moderate (PR, 1.16; 95% CI, 1.06–1.27) and highest (PR, 1.15; 95% CI, 1.05–1.27) NLT-MVPA groups showed significantly higher PRs for high perceived stress in Model 2 (*P* for trend = 0.004). The total MVPA was not significantly associated with high perceived stress in any of the models.

**Table 4:** PRs (95% Cis) of LT-MVPA, NLT-MVPA, and total MVPA for high perceived stress in women.

Variables	Number of cases per participants	Prevalence (%)	Crude				Model 1				Model 2		
			PR	(95% CI)	*P*		PR	(95% CI)	*P*		PR	(95% CI)	*P*
LT-MVPA categories (MET-hours/day)													
Lowest (0)	547/1468	37.3	1.00	(Ref.)			1.00	(Ref.)			1.00	(Ref.)	
Lower-moderate (0.01–1.49)	1207/4115	29.3	0.79	(0.73–0.85)	< 0.001		0.88	(0.81–0.95)	0.002		0.88	(0.81–0.95)	0.002
Upper-moderate (1.50–3.99)	565/2259	25.0	0.67	(0.61–0.74)	< 0.001		0.80	(0.72–0.88)	< 0.001		0.80	(0.72–0.88)	< 0.001
Highest (≥ 4.0)	340/1469	23.1	0.62	(0.55–0.70)	< 0.001		0.79	(0.70–0.89)	< 0.001		0.78	(0.69–0.87)	< 0.001
	2659/9311	28.6	Trend		< 0.001		Trend		0.006		Trend		0.001
NLT-MVPA categories (MET-hours/day)													
Lowest (0-4.9)	1007/3598	28.0	1.00	(Ref.)			1.00	(Ref.)			1.00	(Ref.)	
Lower-moderate (5.0-9.9)	662/2344	28.2	1.01	(0.93–1.10)	0.831		1.04	(0.96–1.13)	0.359		1.06	(0.97–1.15)	0.186
Upper-moderate (10.0-19.9)	508/1711	29.7	1.06	(0.97–1.16)	0.197		1.13	(1.03–1.23)	0.007		1.16	(1.06–1.27)	0.001
Highest (≥ 20.0)	482/1658	29.1	1.04	(0.95–1.14)	0.417		1.11	(1.02–1.22)	0.019		1.15	(1.05–1.27)	0.002
	2659/9311	28.6	Trend		0.476		Trend		0.028		Trend		0.004
Total MVPA categories (MET-hours/day)													
Lowest (0-4.9)	784/2677	29.3	1.00	(Ref.)			1.00	(Ref.)			1.00	(Ref.)	
Lower-moderate (5.0-9.9)	601/2102	28.6	0.98	(0.89–1.07)	0.600		1.04	(0.95–1.14)	0.376		1.04	(0.95–1.14)	0.395
Upper-moderate (10.0-19.9)	685/2440	28.1	0.96	(0.88–1.05)	0.338		1.06	(0.97–1.15)	0.207		1.06	(0.97–1.15)	0.219
Highest (≥ 20.0)	589/2092	28.2	0.96	(0.88–1.05)	0.392		1.08	(0.99–1.18)	0.092		1.08	(0.99–1.18)	0.098
	2659/9311	28.6	Trend		0.452		Trend		0.165		Trend		0.168

Model 1: Adjusted for age, year of health check-up, area of residence, living situation, marital status, educational attainment, body mass index, hypertension, dyslipidemia, heart disease, stroke, diabetes mellitus, cancer, alcohol drinking status, smoking status, and activities of daily living limitation.

Model 2: Adjusted for variables in Model 1 plus sedentary time. For LT-MVPA, further adjusted for NLT-MVPA. For NLT-MVPA, further adjusted for LT-MVPA.

CI, confidence interval; LT, leisure-time; METs, metabolic equivalents; MVPA, moderate-to-vigorous physical activity; NLT, non-leisure-time; PR, prevalence ratio.

The results of the sensitivity analysis were consistent with those of the primary analysis (Supplementary Table 4). Stratified analyses revealed similar results to those of the primary analysis among participants aged 40–64 years (Supplementary Table 5), whereas the associations between the upper-moderate and highest NLT-MVPA groups and high perceived stress were attenuated (Supplementary Table 6).

**Fig. 1:**
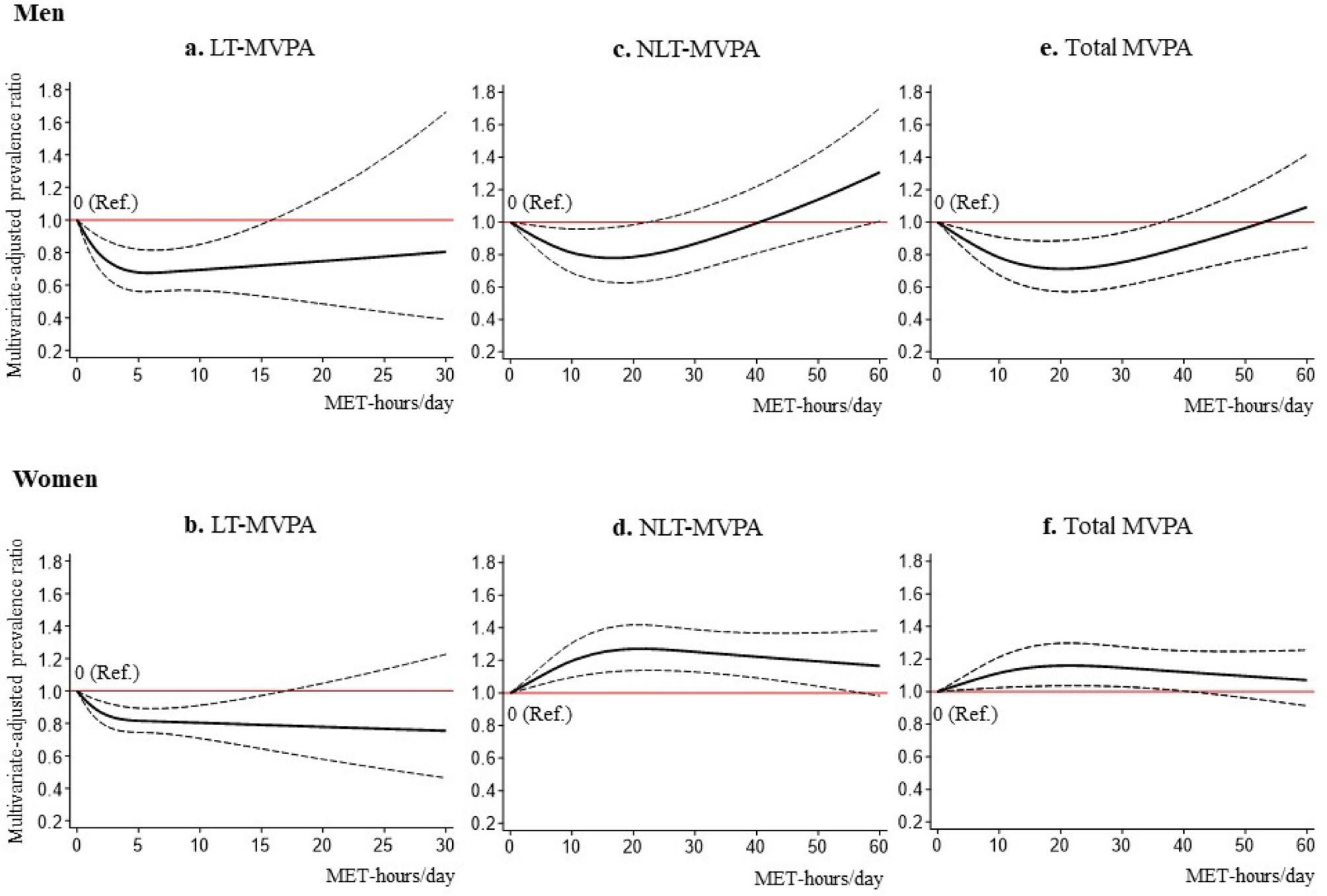
Dose–response associations of domain-specific MVPA with high perceived stress. This figure shows the associations of LT-MVPA (**a**, **b**), NLT-MVPA (**c**, **d**), and total MVPA (**e**, **f**) with high perceived stress modeled by restricted cubic splines. Models were adjusted for age, year of health check-up, area of residence, living situation, marital status, educational attainment, body mass index, hypertension, dyslipidemia, heart disease, stroke, diabetes mellitus, cancer, alcohol drinking status, smoking status, activities of daily living limitation, and sedentary time. Either NLT-MVPA or LT-MVPA was additionally included as a covariate, depending on the independent variable, except when total MVPA was used as the independent variable. The reference value for each model was 0 MET-hours/day. The solid lines indicate the prevalence ratios for high perceived stress. The dashed lines indicate the 95% confidence intervals. LT, leisure-time; METs, metabolic equivalents; MVPA, moderate-to-vigorous physical activity; NLT, non-leisure-time.

Figure 1 illustrates the results of the primary dose–response analyses between LT-MVPA, NLT-MVPA, or total MVPA and high perceived stress in Model 2. For LT-MVPA, compared with no LT-MVPA, the PR for high perceived stress decreased up to approximately 3 MET-hours/day and plateaued at higher levels of LT-MVPA in both sexes (Fig. 1a and b). For NLT-MVPA, the PR for high perceived stress in men declined linearly up to approximately 8 MET-hours/day; however, the association was no longer statistically significant at ≥ 22 MET-hours/day (Fig. 1c). Among women, the PR increased with NLT-MVPA up to approximately 15 MET-hours/day and remained elevated at higher levels (Fig. 1d). For total MVPA, the association pattern reflected a combination of those observed for LT-MVPA and NLT-MVPA in both sexes, but more closely resembled that of NLT-MVPA (Fig. 1e and f).

In age-stratified analyses, participants aged 40–64 years showed association patterns for LT-MVPA, NLT-MVPA, and total MVPA that were consistent with those in the primary analysis (Supplementary Fig. 1). Among those aged 65–74 years, the association patterns for LT-MVPA remained consistent, whereas those for NLT-MVPA and high perceived stress tended to be weaker in both sexes, which was also reflected in the attenuated associations for total MVPA (Supplementary Fig. 2).

## Discussion

The association between MVPA and high perceived stress differed across PA domains. LT-MVPA consistently exhibited an L-shaped association with high perceived stress in both sexes. In contrast, NLT-MVPA showed sex-specific and opposing associations: a J-shaped curve in men and a saturating curve in women. These association patterns were particularly pronounced among participants aged 40–64 years. Our findings suggest that domain- and sex-specific differences should be considered when examining the relationship between MVPA and perceived stress. Increasing LT-MVPA while appropriately regulating NLT-MVPA may be important for effective stress management.

The observed domain-specific differences in the association between MVPA and high perceived stress, along with the L-shaped relationship of LT-MVPA, align with findings from previous studies. A meta-analysis of observational studies^6^ demonstrated that leisure-time PA was positively associated with mental health, while other PA domains were inconsistent. Furthermore, numerous intervention studies^28,29^ have consistently shown that leisure-time PA contributes to improved mental health, including reduced perceived stress. Our findings extend existing evidence by visually demonstrating the differential dose–response curves of LT-MVPA and NLT-MVPA with high perceived stress.

NLT-MVPA, encompassing work, transportation, and household, is often obligatory, repetitive, and lacks perceived control, whereas LT-MVPA is typically self-selected and intrinsically motivated^30^. It may also function as a form of enjoyable distraction, foster a sense of mastery, and facilitate social interaction, thereby enhancing social support^31^, which is a well-established buffer against perceived stress^32^. These contextual differences may help explain the distinct dose–response patterns observed for LT-MVPA and NLT-MVPA in relation to high perceived stress.

A key finding of this study was the sex-specific differences in the shape of the dose–response relationship between NLT-MVPA and high perceived stress. Considering that the work/household domain is the largest contributor to total MVPA^33^ and that transportation-related PA was likely limited among our study participants owing to the predominant use of private vehicles^34^, the observed differences may be attributable to sex-specific variations in sociocultural roles within the work/household domain.

For example, Japanese women traditionally have shorter working hours than men but spend significantly more time on household responsibilities. The National Survey on Family, conducted around the time of our baseline survey^35^, reported that men spent an average of only 31 min per day on housework, compared to 280 min for women. This distinct pattern partly reflects deeply rooted social norms and gender roles in Japan^36^, where even employed women frequently bear a disproportionate burden of household tasks compared to men^35^. This imbalance is also evident in the PA patterns of middle-aged and older Japanese women, who engage in more light PA (LPA) and short-bout MVPA and spend less time in sedentary behavior than men^37,38^. Thus, activities that are particularly common among women, such as household responsibilities in addition to work, may themselves be sources of stress. This may be reflected in the saturating curve observed in the association between NLT-MVPA and high perceived stress among women.

In contrast, occupational PA is likely the primary contributor to NLT-MVPA among men^35^. This interpretation is further supported by the lack of a significant association between NLT-MVPA and high perceived stress in the 65–74 years age group, where many individuals are retired (Supplementary Fig. 2). Physically demanding jobs, such as those in agriculture, forestry, fisheries, construction, and transportation, are more prevalent among men and frequently involve MVPA^39^. These factors may help explain the sex-specific patterns observed in the association with high perceived stress.

Interestingly, the association between NLT-MVPA and high perceived stress among men followed a J-shaped curve in our study. This suggests that low to moderate levels of NLT-MVPA may confer stress-relieving benefits for men, comparable to those of LT-MVPA. In contrast, the association between NLT-MVPA and higher perceived stress may be explained by the Job Demand-Control model^40^. According to this model, the combination of high job demands and low job control, such as limited autonomy over work tasks or decisions, can contribute to elevated stress among workers. Physically demanding jobs often share these characteristics, which may explain the J-shaped association observed in men. Although our data did not capture detailed information on occupational PA and job type, our findings nonetheless contributed valuable insights to the ongoing debate on the “PA paradox” and highlight the importance of considering the shape of the dose–response curve in such analyses.

From a public health perspective, promoting even small increases in LT-MVPA may serve as an effective strategy for stress management. This is particularly relevant for individuals with high levels of NLT-MVPA, who often have low levels of leisure-time PA and may mistakenly consider their overall PA levels sufficient^41^. Previous studies have highlighted the importance of tailored leisure-time PA interventions based on different levels of occupational PA^42^. For example, feasible and low-burden strategies such as “exercise snacking”—short bouts of PA incorporated during breaks or between tasks^43^—may be particularly suitable for individuals with high levels of NLT-MVPA. Implementing exercise snacking programs that specifically target mental health, relaxation, and the alleviation of psychological and physical distress may also play a critical role in comprehensive stress management.

This study is the first to demonstrate the dose–response curves between LT-MVPA and NLT-MVPA and high perceived stress. However, several limitations should be acknowledged. First, there is a possibility of selection bias. Despite a relatively high health check-up attendance rate, participants may represent a more health-conscious population. Second, the cross-sectional design precludes causal inference. As perceived stress may also influence PA behavior^44^, longitudinal studies are warranted to clarify the directionality of this association. Third, although single-item measures of perceived stress have been validated^45,46^ and are widely used in epidemiological studies, the incorporation of objective stress biomarkers may provide additional insights into the biological mechanisms underlying the associations between domain-specific MVPA and stress. In addition, although stress may be influenced by nutritional status^47^, this study did not account for nutritional status or nutrient intake, nor for related physiological and biochemical factors that may act as potential confounders^48,49^. Finally, MVPA was assessed using a single-point self-reported questionnaire, which may have introduced recall bias and misclassification^50^. NLT-MVPA could not be further stratified into specific domains such as occupational, transportation, and household activities. Future studies are warranted to more comprehensively examine the relationships between objectively assessed MVPA, its specific domains, and both subjective and objective measures of stress.

## Conclusions

The dose–response relationship between MVPA and high perceived stress was both domain- and sex-specific. LT-MVPA exhibited a robust L-shaped association with high perceived stress in both sexes. In contrast, NLT-MVPA demonstrated divergent patterns, with a J-shaped curve observed in men and a saturating curve in women. These findings underscore the importance of developing stress management strategies that account for both the domain and sex-specific context of PA.

## Supplementary Information

Below is the link to the electronic supplementary material.

Supplementary Material 1

## Data Availability

The datasets generated during and/or analyzed during the current study are available from the corresponding author on reasonable request.
