# Supplementary material for: Differential dose–response associations of leisure-time and non-leisure-time physical activity with perceived stress in middle-aged and older adults
Source: Sci Rep. 2026 May 12;16:21751. doi: 10.1038/s41598-026-52997-4 (PMC13358078; doi:10.1038/s41598-026-52997-4)
Supplement: Supplementary file 1 — Supplementary Material 1 [file 41598_2026_52997_MOESM1_ESM.pdf]

Supplementary Table 1. PRs of domain-specific MVPA for high perceived stress with multiple imputation of covariates in men

Variables	Number of cases per participants	Prevalence (%)	Crude			Model 1			Model 2		
			PR	(95% CI)	*P*	PR	(95% CI)	*P*	PR	(95% CI)	*P*
**LT-MVPA categories (MET-hours/day)**											
Lowest (0)	198/1022	19.4	1.00	(Ref.)		1.00	(Ref.)		1.00	(Ref.)	
Lower-moderate (0.01-1.49)	329/2310	14.2	0.74	(0.63-0.86)	<0.001	0.87	(0.74-1.02)	0.084	0.87	(0.74-1.02)	0.090
Upper-moderate (1.50-3.99)	159/1578	10.1	0.52	(0.43-0.63)	<0.001	0.71	(0.58-0.87)	0.001	0.71	(0.58-0.87)	0.001
Highest (≥4.0)	130/1467	8.9	0.46	(0.37-0.56)	<0.001	0.68	(0.55-0.84)	<0.001	0.68	(0.55-0.84)	<0.001
	816/6377	12.8	Trend		<0.001	Trend		0.023	Trend		0.017
**NLT-MVPA categories (MET-hours/day)**											
Lowest (0-4.9)	368/2534	14.5	1.00	(Ref.)		1.00	(Ref.)		1.00	(Ref.)	
Lower-moderate (5.0-9.9)	132/1200	11.0	0.76	(0.63-0.91)	0.004	0.85	(0.71-1.02)	0.088	0.88	(0.74-1.06)	0.192
Upper-moderate (10.0-19.9)	140/1311	10.7	0.74	(0.61-0.88)	0.001	0.77	(0.64-0.93)	0.006	0.82	(0.68-0.99)	0.036
Highest (≥20.0)	176/1332	13.2	0.91	(0.77-1.08)	0.267	0.95	(0.80-1.12)	0.545	1.03	(0.87-1.22)	0.723
	816/6377	12.8	Trend		0.836	Trend		0.671	Trend		0.173
**Total MVPA categories (MET-hours/day)**											
Lowest (0-4.9)	251/1605	15.6	1.00	(Ref.)		1.00	(Ref.)		1.00	(Ref.)	
Lower-moderate (5.0-9.9)	173/1267	13.7	0.87	(0.73-1.04)	0.138	1.01	(0.84-1.20)	0.944	1.01	(0.85-1.20)	0.908
Upper-moderate (10.0-19.9)	191/1801	10.6	0.68	(0.57-0.81)	<0.001	0.79	(0.66-0.94)	0.008	0.80	(0.67-0.96)	0.016

Highest (≥20.0)	201/1704	11.8	0.75 (0.63-0.90)	0.001	0.87 (0.73-1.03)	0.111	0.91 (0.76-1.08)	0.276
	816/6377	12.8	Trend	0.228	Trend	0.740	Trend	0.852

Model 1: Adjusted for age, year of health check-up, area of residence, living situation, marital status, educational attainment, body mass index, hypertension, dyslipidemia, heart disease, stroke, diabetes mellitus, cancer, alcohol drinking status, smoking status, and activities of daily living limitation.

Model 2: Adjusted for variables in Model 1 plus sedentary time. For LT-MVPA, further adjusted for NLT-MVPA. For NLT-MVPA, further adjusted for LT-MVPA.

CI, confidence interval; LT, leisure-time; METs, metabolic equivalents; MVPA, moderate-to-vigorous physical activity; NLT, non-leisure-time; PR, prevalence ratio.

Supplementary Table 2. PRs of domain-specific MVPA for high perceived stress in men aged 40-64 years

Variables	Number of cases per participants	Prevalence (%)	Crude			Model 1			Model 2		
			PR	(95% CI)	*P*	PR	(95% CI)	*P*	PR	(95% CI)	*P*
**LT-MVPA categories (MET-hours/day)**											
Lowest (0)	152/638	23.8	1.00	(Ref.)		1.00	(Ref.)		1.00	(Ref.)	
Lower-moderate (0.01-1.49)	220/1097	20.1	0.84	(0.70-1.01)	0.064	0.91	(0.76-1.09)	0.295	0.92	(0.77-1.10)	0.351
Upper-moderate (1.50-3.99)	96/560	17.1	0.72	(0.57-0.90)	0.005	0.84	(0.67-1.06)	0.145	0.85	(0.67-1.07)	0.168
Highest (≥4.0)	52/408	12.8	0.53	(0.40-0.71)	<0.001	0.66	(0.49-0.88)	0.004	0.66	(0.49-0.88)	0.005
	520/2703	19.2	Trend		0.021	Trend		0.165	Trend		0.142
**NLT-MVPA categories (MET-hours/day)**											
Lowest (0-4.9)	238/1107	21.5	1.00	(Ref.)		1.00	(Ref.)		1.00	(Ref.)	
Lower-moderate (5.0-9.9)	75/449	16.7	0.78	(0.61-0.98)	0.036	0.84	(0.67-1.06)	0.142	0.87	(0.69-1.10)	0.252
Upper-moderate (10.0-19.9)	81/545	14.9	0.69	(0.55-0.87)	0.002	0.70	(0.55-0.89)	0.003	0.73	(0.57-0.93)	0.010
Highest (≥20.0)	126/602	20.9	0.97	(0.80-1.18)	0.784	1.01	(0.83-1.23)	0.905	1.06	(0.86-1.30)	0.597
	520/2703	19.2	Trend		0.582	Trend		0.307	Trend		0.139
**Total MVPA categories (MET-hours/day)**											
Lowest (0-4.9)	172/780	22.1	1.00	(Ref.)		1.00	(Ref.)		1.00	(Ref.)	
Lower-moderate (5.0-9.9)	106/500	21.2	0.96	(0.78-1.19)	0.719	1.05	(0.85-1.30)	0.655	1.06	(0.86-1.31)	0.586
Upper-moderate (10.0-19.9)	108/704	15.3	0.70	(0.56-0.87)	0.001	0.75	(0.60-0.94)	0.012	0.77	(0.62-0.96)	0.021
Highest (≥20.0)	134/719	18.6	0.85	(0.69-1.03)	0.102	0.92	(0.75-1.14)	0.451	0.95	(0.76-1.17)	0.622

520/2703	19.2	Trend	0.801	Trend	0.645	Trend	0.451

Model 1: Adjusted for age, year of health check-up, area of residence, living situation, marital status, educational attainment, body mass index, hypertension, dyslipidemia, heart disease, stroke, diabetes mellitus, cancer, alcohol drinking status, smoking status, and activities of daily living limitation.

Model 2: Adjusted for variables in Model 1 plus sedentary time. For LT-MVPA, further adjusted for NLT-MVPA. For NLT-MVPA, further adjusted for LT-MVPA.

CI, confidence interval; LT, leisure-time; METs, metabolic equivalents; MVPA, moderate-to-vigorous physical activity; NLT, non-leisure-time; PR, prevalence ratio.

Supplementary Table 3. PRs of domain-specific MVPA for high perceived stress in men aged 65-74 years

Variables	Number of cases per participants	Prevalence (%)	Crude			Model 1			Model 2		
			PR	(95% CI)	*P*	PR	(95% CI)	*P*	PR	(95% CI)	*P*
**LT-MVPA categories (MET-hours/day)**											
Lowest (0)	46/384	12.0	1.00	(Ref.)		1.00	(Ref.)		1.00	(Ref.)	
Lower-moderate (0.01-1.49)	109/1213	9.0	0.75	(0.54-1.04)	0.083	0.76	(0.54-1.05)	0.096	0.75	(0.54-1.04)	0.082
Upper-moderate (1.50-3.99)	63/1018	6.2	0.52	(0.36-0.74)	<0.001	0.54	(0.37-0.78)	0.001	0.52	(0.36-0.76)	0.001
Highest (≥4.0)	78/1059	7.4	0.61	(0.44-0.87)	0.006	0.64	(0.45-0.92)	0.015	0.65	(0.45-0.93)	0.019
	296/3674	8.1	Trend		0.039	Trend		0.068	Trend		0.089
**NLT-MVPA categories (MET-hours/day)**											
Lowest (0-4.9)	130/1427	9.1	1.00	(Ref.)		1.00	(Ref.)		1.00	(Ref.)	
Lower-moderate (5.0-9.9)	57/751	7.6	0.83	(0.62-1.12)	0.231	0.86	(0.63-1.16)	0.313	0.87	(0.64-1.19)	0.389
Upper-moderate (10.0-19.9)	59/766	7.7	0.85	(0.63-1.14)	0.265	0.86	(0.64-1.16)	0.318	0.94	(0.69-1.27)	0.675
Highest (≥20.0)	50/730	6.9	0.75	(0.55-1.03)	0.075	0.79	(0.57-1.09)	0.146	0.89	(0.64-1.24)	0.508
	296/3674	8.1	Trend		0.207	Trend		0.331	Trend		0.843
**Total MVPA categories (MET-hours/day)**											
Lowest (0-4.9)	79/825	9.6	1.00	(Ref.)		1.00	(Ref.)		1.00	(Ref.)	
Lower-moderate (5.0-9.9)	67/767	8.7	0.91	(0.67-1.24)	0.562	0.95	(0.70-1.31)	0.773	0.95	(0.69-1.30)	0.729
Upper-moderate (10.0-19.9)	83/1097	7.6	0.79	(0.59-1.06)	0.117	0.83	(0.61-1.12)	0.227	0.84	(0.62-1.13)	0.243
Highest (≥20.0)	67/985	6.8	0.71	(0.52-0.97)	0.032	0.75	(0.54-1.04)	0.082	0.79	(0.56-1.10)	0.157

	296/3674	8.1	Trend	0.088	Trend	0.166	Trend	0.302

Model 1: Adjusted for age, year of health check-up, area of residence, living situation, marital status, educational attainment, body mass index, hypertension, dyslipidemia, heart disease, stroke, diabetes mellitus, cancer, alcohol drinking status, smoking status, and activities of daily living limitation.

Model 2: Adjusted for variables in Model 1 plus sedentary time. For LT-MVPA, further adjusted for NLT-MVPA. For NLT-MVPA, further adjusted for LT-MVPA.

CI, confidence interval; LT, leisure-time; METs, metabolic equivalents; MVPA, moderate-to-vigorous physical activity; NLT, non-leisure-time; PR, prevalence ratio.

Supplementary Table 4. PRs of domain-specific MVPA for high perceived stress with multiple imputation of covariates in women

Variables	Number of cases per participants	Prevalence (%)	Crude			Model 1			Model 2		
			PR	(95% CI)	*P*	PR	(95% CI)	*P*	PR	(95% CI)	*P*
**LT-MVPA categories (MET-hours/day)**											
Lowest (0)	547/1468	37.3	1.00	(Ref.)		1.00	(Ref.)		1.00	(Ref.)	
Lower-moderate (0.01-1.49)	1207/4115	29.3	0.79	(0.73-0.85)	<0.001	0.88	(0.81-0.95)	0.002	0.88	(0.81-0.96)	0.002
Upper-moderate (1.50-3.99)	565/2259	25.0	0.67	(0.61-0.74)	<0.001	0.80	(0.72-0.88)	<0.001	0.80	(0.72-0.88)	<0.001
Highest (≥4.0)	340/1469	23.1	0.62	(0.55-0.70)	<0.001	0.79	(0.71-0.89)	<0.001	0.78	(0.69-0.87)	<0.001
	2659/9311	28.6	Trend		<0.001	Trend		0.006	Trend		0.001
**NLT-MVPA categories (MET-hours/day)**											
Lowest (0-4.9)	1007/3598	28.0	1.00	(Ref.)		1.00	(Ref.)		1.00	(Ref.)	
Lower-moderate (5.0-9.9)	662/2344	28.2	1.01	(0.93-1.10)	0.831	1.04	(0.96-1.13)	0.347	1.06	(0.97-1.15)	0.180
Upper-moderate (10.0-19.9)	508/1711	29.7	1.06	(0.97-1.16)	0.197	1.13	(1.04-1.24)	0.006	1.16	(1.06-1.27)	0.001
Highest (≥20.0)	482/1658	29.1	1.04	(0.95-1.14)	0.417	1.11	(1.02-1.2)	0.019	1.15	(1.05-1.27)	0.002
	2659/9311	28.6	Trend		0.476	Trend		0.027	Trend		0.003
**Total MVPA categories (MET-hours/day)**											
Lowest (0-4.9)	784/2677	29.3	1.00	(Ref.)		1.00	(Ref.)		1.00	(Ref.)	
Lower-moderate (5.0-9.9)	601/2102	28.6	0.98	(0.89-1.07)	0.600	1.04	(0.95-1.14)	0.381	1.04	(0.95-1.13)	0.400
Upper-moderate (10.0-19.9)	685/2440	28.1	0.96	(0.88-1.05)	0.338	1.06	(0.97-1.15)	0.202	1.06	(0.97-1.15)	0.214
Highest (≥20.0)	589/2092	28.2	0.96	(0.88-1.05)	0.392	1.08	(0.99-1.18)	0.093	1.08	(0.99-1.18)	0.098

2659/9311	28.6	Trend	0.452	Trend	0.160	Trend	0.163

Model 1: Adjusted for age, year of health check-up, area of residence, living situation, marital status, educational attainment, body mass index, hypertension, dyslipidemia, heart disease, stroke, diabetes mellitus, cancer, alcohol drinking status, smoking status, and activities of daily living limitation.

Model 2: Adjusted for variables in Model 1 plus sedentary time. For LT-MVPA, further adjusted for NLT-MVPA. For NLT-MVPA, further adjusted for LT-MVPA.

CI, confidence interval; LT, leisure-time; METs, metabolic equivalents; MVPA, moderate-to-vigorous physical activity; NLT, non-leisure-time; PR, prevalence ratio.

Supplementary Table 5. PRs of domain-specific MVPA for high perceived stress in women aged 40-64 years

Variables	Number of cases per participants	Prevalence (%)	Crude			Model 1			Model 2		
			PR	(95% CI)	*P*	PR	(95% CI)	*P*	PR	(95% CI)	*P*
**LT-MVPA categories (MET-hours/day)**											
Lowest (0)	463/1092	42.4	1.00	(Ref.)		1.00	(Ref.)		1.00	(Ref.)	
Lower-moderate (0.01-1.49)	902/2603	34.7	0.82	(0.75-0.89)	<0.001	0.86	(0.79-0.94)	0.001	0.87	(0.79-0.95)	0.001
Upper-moderate (1.50-3.99)	370/1200	30.8	0.73	(0.65-0.81)	<0.001	0.79	(0.71-0.88)	<0.001	0.79	(0.71-0.89)	<0.001
Highest (≥4.0)	202/669	30.2	0.71	(0.62-0.81)	<0.001	0.79	(0.69-0.91)	0.001	0.78	(0.68-0.89)	<0.001
	1937/5564	34.8	Trend		<0.001	Trend		0.007	Trend		0.002
**NLT-MVPA categories (MET-hours/day)**											
Lowest (0-4.9)	749/2193	34.2	1.00	(Ref.)		1.00	(Ref.)		1.00	(Ref.)	
Lower-moderate (5.0-9.9)	478/1425	33.5	0.98	(0.85-1.12)	0.705	1.01	(0.92-1.11)	0.802	1.03	(0.94-1.14)	0.487
Upper-moderate (10.0-19.9)	353/967	36.5	1.07	(0.95-1.30)	0.198	1.11	(1.01-1.23)	0.039	1.14	(1.03-1.26)	0.013
Highest (≥20.0)	357/979	36.5	1.07	(0.95-1.29)	0.204	1.13	(1.02-1.25)	0.016	1.17	(1.05-1.30)	0.003
	1937/5564	34.8	Trend		0.174	Trend		0.016	Trend		0.003
**Total MVPA categories (MET-hours/day)**											
Lowest (0-4.9)	610/1730	35.3	1.00	(Ref.)		1.00	(Ref.)		1.00	(Ref.)	
Lower-moderate (5.0-9.9)	430/1256	34.2	0.97	(0.88-1.07)	0.562	1.01	(0.91-1.12)	0.842	1.01	(0.92-1.12)	0.834
Upper-moderate (10.0-19.9)	476/1395	34.1	0.97	(0.88-1.07)	0.507	1.03	(0.93-1.13)	0.588	1.03	(0.93-1.13)	0.612
Highest (≥20.0)	421/1183	35.6	1.01	(0.91-1.12)	0.856	1.09	(0.98-1.20)	0.098	1.09	(0.98-1.20)	0.111

1937/5564	34.8	Trend	0.716	Trend	0.099	Trend	0.117

Model 1: Adjusted for age, year of health check-up, area of residence, living situation, marital status, educational attainment, body mass index, hypertension, dyslipidemia, heart disease, stroke, diabetes mellitus, cancer, alcohol drinking status, smoking status, and activities of daily living limitation.

Model 2: Adjusted for variables in Model 1 plus sedentary time. For LT-MVPA, further adjusted for NLT-MVPA. For NLT-MVPA, further adjusted for LT-MVPA.

CI, confidence interval; LT, leisure-time; METs, metabolic equivalents; MVPA, moderate-to-vigorous physical activity; NLT, non-leisure-time; PR, prevalence ratio.

Supplementary Table 6. PRs of domain-specific MVPA for high perceived stress in women aged 65-74 years

Variables	Number of cases per participants	Prevalence (%)	Crude			Model 1			Model 2		
			PR	(95% CI)	*P*	PR	(95% CI)	*P*	PR	(95% CI)	*P*
**LT-MVPA categories (MET-hours/day)**											
Lowest (0)	84/376	22.3	1.00	(Ref.)		1.00	(Ref.)		1.00	(Ref.)	
Lower-moderate (0.01-1.49)	305/1512	20.2	0.90	(0.73-1.12)	0.349	0.92	(0.74-1.13)	0.424	0.92	(0.74-1.14)	0.432
Upper-moderate (1.50-3.99)	195/1059	18.4	0.82	(0.66-1.03)	0.095	0.81	(0.65-1.02)	0.078	0.81	(0.65-1.02)	0.070
Highest (≥4.0)	138/800	17.3	0.77	(0.61-0.98)	0.036	0.81	(0.63-1.03)	0.089	0.81	(0.63-1.03)	0.088
	722/3747	19.3	Trend		0.221	Trend		0.372	Trend		0.359
**NLT-MVPA categories (MET-hours/day)**											
Lowest (0-4.9)	258/1405	18.4	1.00	(Ref.)		1.00	(Ref.)		1.00	(Ref.)	
Lower-moderate (5.0-9.9)	184/919	20.0	1.09	(0.92-1.29)	0.318	1.09	(0.92-1.29)	0.333	1.10	(0.92-1.30)	0.287
Upper-moderate (10.0-19.9)	155/744	20.8	1.13	(0.95-1.36)	0.165	1.17	(0.98-1.39)	0.092	1.19	(0.99-1.43)	0.059
Highest (≥20.0)	125/679	18.4	1.00	(0.83-1.22)	0.980	1.04	(0.85-1.26)	0.723	1.07	(0.87-1.31)	0.541
	722/3747	19.3	Trend		0.723	Trend		0.983	Trend		0.775
**Total MVPA categories (MET-hours/day)**											
Lowest (0-4.9)	174/947	18.4	1.00	(Ref.)		1.00	(Ref.)		1.00	(Ref.)	
Lower-moderate (5.0-9.9)	171/846	20.2	1.10	(0.91-1.33)	0.324	1.11	(0.91-1.34)	0.303	1.10	(0.91-1.33)	0.327
Upper-moderate (10.0-19.9)	209/1045	20.0	1.09	(0.91-1.30)	0.358	1.12	(0.93-1.34)	0.232	1.11	(0.93-1.34)	0.244
Highest (≥20.0)	168/909	18.5	1.01	(0.83-1.22)	0.952	1.04	(0.86-1.27)	0.670	1.04	(0.85-1.27)	0.690

722/3747	19.3	Trend	0.537	Trend	0.854	Trend	0.863

Model 1: Adjusted for age, year of health check-up, area of residence, living situation, marital status, educational attainment, body mass index, hypertension, dyslipidemia, heart disease, stroke, diabetes mellitus, cancer, alcohol drinking status, smoking status, and activities of daily living limitation.

Model 2: Adjusted for variables in Model 1 plus sedentary time. For LT-MVPA, further adjusted for NLT-MVPA. For NLT-MVPA, further adjusted for LT-MVPA.

CI, confidence interval; LT, leisure-time; METs, metabolic equivalents; MVPA, moderate-to-vigorous physical activity; NLT, non-leisure-time; PR, prevalence ratio.

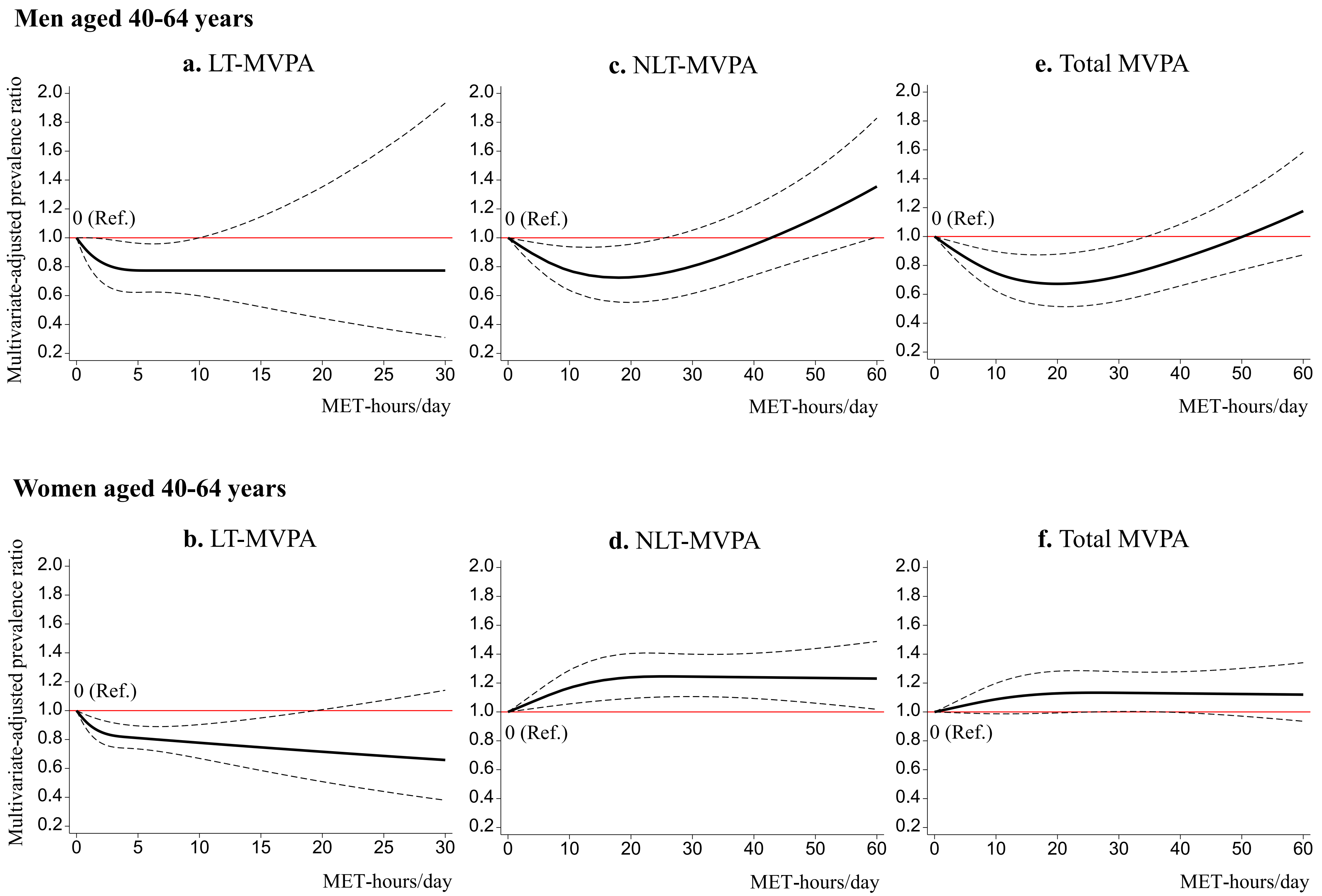

Supplementary Figure. 1.
Dose–response associations of domain-specific MVPA with high perceived stress among adults aged 40-64 years

Supplementary Fig. 1. Dose–response associations of domain-specific MVPA with high perceived stress among adults aged 40–64 years

This figure shows the associations of LT-MVPA (Supplementary Figure 1a, b), NLT-MVPA (Supplementary Figure 1c, d), and total MVPA (Supplementary Figure 1e, f) with high perceived stress among adults aged 40–64 years modeled by restricted cubic splines. Models were adjusted for age, year of health check-up, area of residence, living situation, marital status, educational attainment, body mass index, hypertension, dyslipidemia, heart disease, stroke, diabetes mellitus, cancer, alcohol drinking status, smoking status, activities of daily living limitation, and sedentary time. Either NLT-MVPA or LT-MVPA was additionally included as a covariate, depending on the independent variable, except when total MVPA was used as the independent variable. The reference value for each model was 0 MET MET-hours/day. The solid lines indicate the prevalence ratios for high perceived stress. The dashed lines indicate the 95% confidence intervals. LT, leisure-time; METs, metabolic equivalents; MVPA, moderate-to-vigorous physical activity; NLT, non-leisure-time.

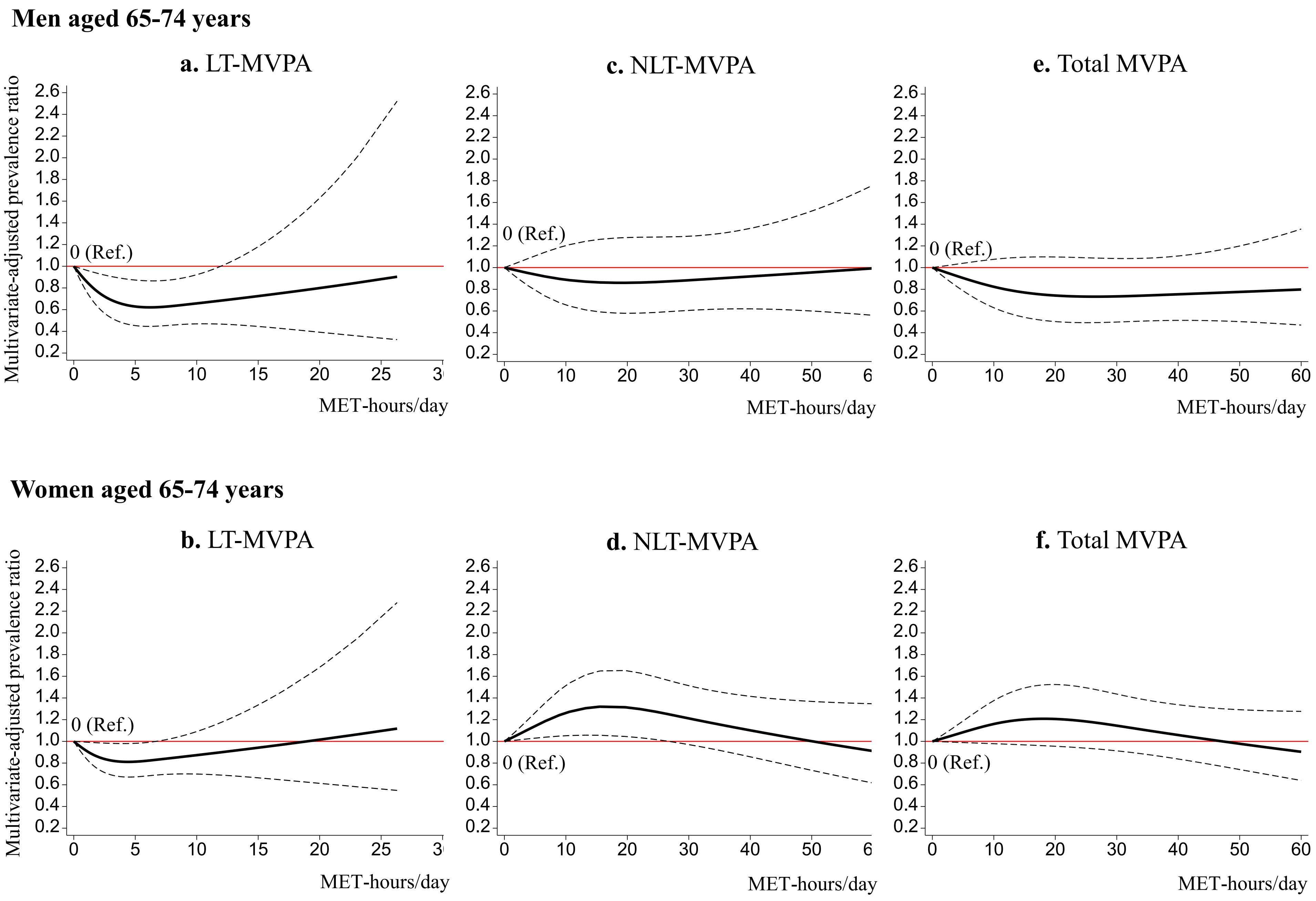

Supplementary Figure. 2.
Dose-response associations of domain-specific MVPA with high perceived stress among adults aged 65-74 years

Supplementary Fig. 2. Dose–response associations of domain-specific MVPA with high perceived stress among adults aged 65–74 years.

This figure shows the associations of LT-MVPA (Supplementary Figure 2a, b), NLT-MVPA (Supplementary Figure 2c, d), and total MVPA (Supplementary Figure 2e, f) with high perceived stress among adults aged 65–74 years modeled by restricted cubic splines. Models were adjusted for age, year of health check-up, area of residence, living situation, marital status, educational attainment, body mass index, hypertension, dyslipidemia, heart disease, stroke, diabetes mellitus, cancer, alcohol drinking status, smoking status, activities of daily living limitation, and sedentary time. Either NLT-MVPA or LT-MVPA was additionally included as a covariate, depending on the independent variable, except when total MVPA was used as the independent variable. The reference value for each model was 0 MET MET-hours/day. The solid lines indicate the prevalence ratios for high perceived stress. The dashed lines indicate the 95% confidence intervals. LT, leisure-time; METs, metabolic equivalents; MVPA, moderate-to-vigorous physical activity; NLT, non-leisure-time.
